# An efficient and reproducible cryopreservation protocol for sustainable conservation of Jojoba (*Simmondsia chinensis*)

**DOI:** 10.1038/s41598-025-30951-0

**Published:** 2025-12-01

**Authors:** Era Vaidya Malhotra, Suresh Chand Mali, Sangita Bansal, Anju Mahendru Singh

**Affiliations:** ICAR-National Bureau of Plant Genetic Resources, New Delhi, India

**Keywords:** Cryopreservation, Cryobanking, Droplet vitrification, Ex situ conservation, Post-thaw survival, Biological techniques, Biotechnology, Plant sciences

## Abstract

*Simmondsia chinensis*, commonly known as jojoba, is an important renewable source of liquid wax esters valued for its unique seed oil that is used in several industries. Elite jojoba germplasm needs to be conserved owing to its overexploitation, climate variability and excessive dependence of selected cultivars. This study presents a straightforward and efficient droplet vitrification-based cryopreservation protocol in jojoba. Shoot tips isolated from eight-week-old cultures were cultured for two days on high sucrose (0.3 M) enriched medium and then treated with loading solution containing 0.4 M sucrose and 2 M glycerol, followed by PVS2 exposure for 30 min at room temperature. Vitrified shoot tips were then directly frozen in liquid nitrogen by placing them on aluminium foil strips. Frozen shoot tips were rewarmed in an unloading solution containing 1.2 M sucrose for 15 min, and then cultured on regeneration medium consisting of Murashige and Skoog medium (MS) supplemented with 4.97µM benzyl-amino purine (BAP) and 0.28 µM gibberellic acid (GA_3_). The protocol was optimized in one accession, where as high as 85.7% post-thaw survival and 76.1% regrowth were observed. The developed protocol was then tested for its efficacy and reproducibility on eleven other accession, and high post-thaw regrowth, ranging from 50 to 76.13% was observed. This study presents a broad spectrum, reproducible and efficient protocol for the conservation of jojoba genetic resources. This is the first report on cryopreservation of jojoba germplasm for its long-term conservation, providing a technical platform to set up cryobanks of valuable material of this important commercial crop.

## Introduction

Jojoba (*Simmondsia chinensis* (link) Schneider) a dioecious desert shrub native to the Sonoran Desert and Baja California regions of North America, is commercially very valuable for its unusual seed oil that consists primarily of liquid wax esters and is popularly known as green gold. It’s oil is among the three most important non-edible oils, alongside *Jatropha curcas* and *Camelina sativa*, and is widely used in cosmetics, pharmaceuticals as well as industrial and aerospace lubricants^1–3^. As jojoba seeds are a sustainable source of liquid wax esters, they have been used as an eco-friendly alternative for oils harvested from the spermaceti organ of the threatened sperm whale (*Physeter macrocephalus*)^4^. These wax esters are esters of monounsaturated long-chain fatty acid (C_20_-C_24_) and a fatty alcohol (C_20_-C_24_), and are reported to have high compatibility with human sebum, and hence find use in a variety of cosmetic products^5–7^. Jojoba oil is also used in industrial lubricants as it has excellent mechanical lubricity and is stable at high temperatures and pressures and has antifoaming, antiwear, and antirust properties^8–10^. Apart from its industrial uses, jojoba is also known to have medicinal properties and is used by Native Americans as a remedy for cancer, obesity, and throat warts^11,12^.

As jojoba breeding is not an economically viable option, its market depends on elite line multiplied by clonal propagation. Hence it becomes imperative to devise methods to conserve the elite germplasm of this crop for future adaptability and cultivation. To date studies on conservation of jojoba genetic resources are limited. Few reports on medium term conservation of jojoba using slow growth methods are available^13–15^. However, no attempts have been made for the long-term conservation of this plant. Cryopreservation i.e. cryogenic preservation in liquid nitrogen (− 196 °C) is an ideal method for long term conservation of plant genetic resources which has been employed for the conservation of many economically important plant species. At this low temperature, all the metabolic activities metabolic, physical and chemical alterations are arrested and biological material can be stored for hundreds of years without losing their viability^16^. Further, conserving crop germplasm by cryopreservation helps in reducing the maintenance cost as well as the chances of occurrence of genetic and epigenetic variations^17,18^. Cryobanking for conservation of plant genetic resources were initiated during 1970–1980 s to act as backup collections for germplasm conserved in active in vitro as well as field genebanks^19^. Several genebanks across the world now have cryopreserved crop collections, however; approximately 100,000 unique accessions of vegetatively propagated and recalcitrant seed crops are yet to be conserved and protocols for long-term conservation through cryopreservation need to be developed^20,21^. The present study was undertaken to study the effect of vitrification and freezing on jojoba shoot tips by comparing the effects of plant vitrification solutions 2 (PVS2) and PVS3 on shoot regrowth in cryopreserved shoot tips in order to optimize a droplet vitrification technique for cryopreservation of jojoba. The developed protocol was validated on twelve different accessions to confirm its applicability.

## Results

### Recovery of cryopreserved shoot tips

**Fig. 1:**
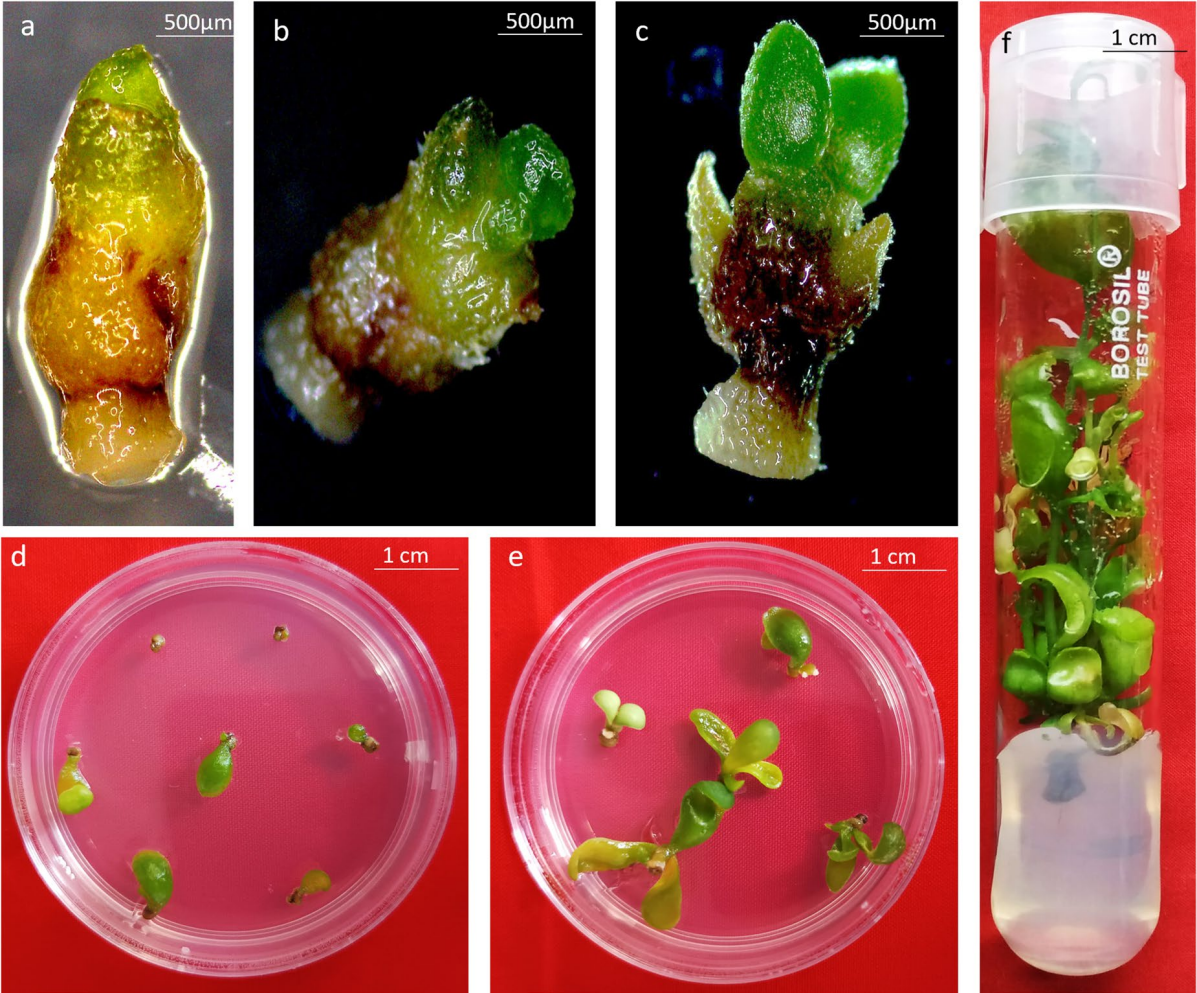
Recovery process of shoot tips after cryostorage; (**a**) Shoot tips excised for cryopreservation, (**b**) a surviving shoot tip after seven days of post-thaw culture following cryopreservation, (**c**) surviving shoot tip with two fully-opened leaves after 20 days of post-thaw culture following cryopreservation, (**d,e**) plantlets recovered from cryopreserved shoot tips after 6 weeks and 10 weeks, (**f**) well developed plantlet regenerated from cryopreserved shoot tips.

Shoot tips surviving cryostorage turned green within seven days of post-cryo rewarming (Fig. 1b) followed by regrowth characterized by unfolding of small green leaves within 20 days (Fig. 1c). The non-surviving shoot tips turned brown. The shoot tips were allowed to grow on the regeneration medium for eight weeks, following which they were transferred to the maintenance medium where they grew into small, well developed, healthy plantlets (Fig. 1d–f).

### Effect of preculture duration on recovery of cryopreserved shoot tips

**Fig. 2:**
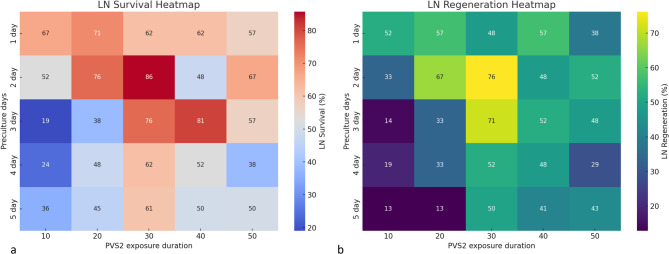
Post thaw survival (**a**) and regeneration (**b**) across different PVS2 exposure durations and preculture days. X-axis: PVS2 exposure duration; Y-axis: Preculture days Brighter shades indicate higher values.

All shoot tips precultured on PCM for 1–5 days survived and regenerated. A significant effect of preculture duration on post-thaw survival and regrowth was observed. Survival of cryopreserved shoot tips (+ LN) ranged from 60 to 85%; shoot tips precultured on PCM for 2 days followed by cryoprotection with PVS2 (30 min) exhibited the highest survival, i.e. 86% which gradually decreased with increase in preculture duration (Fig. 2). The pattern of regrowth followed a similar trend, with 76% post-thaw regrowth observed in shoot tips precultured for 2 days and a decline in regrowth percent thereafter (Fig. 2). Thus, a 2-day preculture was found to be optimal and used for further experiments.

### Effect of PVS2 exposure on post-thaw regrowth of cryopreserved shoot tips

**Fig. 3:**
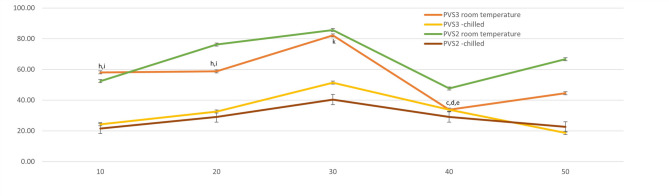
Post that survival (**a**) and regeneration (**b**) of shoot tips across cryoprotectant types and exposure durations. Each line represents a treatment condition. Data is presented as mean ± SE.

All the treated control shoot tips (-LN) showed a similar high level of regrowth (80–100%) when exposed to PVS2 at room temperature as well as at 0 °C for durations ranging from 10 to 50 min (Fig. 3). Highest post-thaw shoot tip survival (85.7%) as well as regrowth (76.19%) was recorded at 30 min PVS2 treatment at room temperature, which then gradually decreased as the PVS2 exposure duration increased further. Although, a slight improvement was observed at 50 min in both survival and recovery (67% and 52%, respectively), it did not reach close to those observed at 30 min exposure. This overall trend indicated that prolonged exposure to PVS2 negatively affected both survival as well as post thaw regrowth (Fig. 3). On treating the shoot tips with chilled PVS2, post-thaw survival was found to increase with increasing PVS2 exposure duration upto 30 min. Highest post-thaw survival and regrowth was noted in shoot tips treated with PVS2 for 30 min, and it declined on further increase in PVS2 exposure duration. However, the post-thaw survival and regrowth response was significantly lower to treatment with PVS2 at room temperature (Fig. 3).

The post-thaw regenerated plants were morphologically similar to the in vitro stock plants. The plant regenerated well on the regeneration medium to form fully grown plantlets and no difference was observed between the plant height, leaf morphology and overall plant growth pattern of the in vitro stock plants and the post-thaw regenerated plants.

### Effect of PVS3 exposure on post-thaw regrowth of cryopreserved shoot tips

In case of treatment with PVS3, 30 min exposure at room temperature was found to be the most effective in comparison to other exposure durations, with 71% post-thaw shoot tip survival followed by 50% regrowth (Fig. 3). Shorter as well as longer exposure durations resulted in reduced post-thaw survival and regrowth in comparison to the control and PVS3 30 min treatment. Using chilled PVS3 too resulted in similar observations that shorter and longer exposure durations resulted in reduced post-thaw survival and regrowth with 30 min exposure duration yielding the most optimal results (51% post-thaw survival and regrowth). However, here again it was observed that treatment with PVS3 at room temperature resulted in better and higher survival and regrowth that that observed using chilled PVS3 (Fig. 3).

Post-thaw shoot tip survival and regeneration was observed in all the treatments of both PVS2 and PVS3, however the response was found to be significantly higher in shoot tips treated with PVS2 for 30 min at room temperature (Fig. 3).

### Assessment of genetic stability

**Fig. 4:**
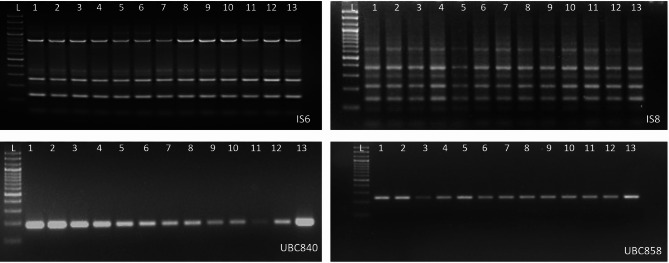
ISSR amplification profile of in vitro stock shoots (1–3), cryopreservation control (-LN) shoot tips (4–7) and post-thaw regenerated cryopreserved (+ LN) shoot tips (8–13) with primers IS6, IS8, UBC840 and UBC858; L − 100 bp Molecular weight marker.

Out of the 30 ISSR markers, 24 (80%) displayed compact, good quality bands. Banding profiles of stock cultures, -LN and + LN plants were compared to confirm their clonal fidelity. A total of 53 bands were scored across all the plants, ranging from 100 to 900 bp (Table 1; Fig. 4). Single amplicons were generated by primers IS11, IS1, UBC840, UBC 858; while the highest number of amplicons was generated by primer IS8. All the primers revealed 100% monomorphism, with no variation detected between of the plants at the tested loci, indicating no induced variations due to the cryopreservation process.

**Table 1:** Primers used for genetic stability assessment.

S. no.	Primer name	Sequence (3’-5’)	Annealing Tm	Number of amplified bands	Band size (bp)
1.	IS6	(GA)_8_C	51.2 °C	3	100–900
2.	IS7	(GT)_8_A	50.7°	2	200, 400
3.	IS8	(AG)_8_C	51.2°	5	100–700
4.	IS9	(TG)_7_TA	53.6°	3	200–500
5.	IS11	(CA)_7_G	52.2°	1	300
6.	IS12	(GT)_8_C	52.2°	1	200
7.	IS53	(AG)_7_C	54.0°	3	200–600
8.	IS61	(GA)_8_T	54.0°	3	200–900
9.	IS65	(AG)_8_T	52.8°	2	200, 500
10.	UBC814	(CT)_8_A	50°	-	–
11.	UBC819	(GT)_8_A	50°	-	–
12.	UBC820	(GT)_8_C	50°	-	–
13.	UBC825	(AC)_8_T	52.8°	2	100, 500
14.	UBC826	(AC)_8_C	52.8°	2	200, 500
15.	UBC828	(TG)_8_A	50°	2	100, 400
16.	UBC834	(AG)_8_CT	50.6°	-	–
17.	UBC835	(AG)_8_TC	50.6°	-	–
18.	UBC836	(AG)_8_TA	50°	-	–
19.	UBC840	(GA)_8_CT	50°	1	200
20.	UBC841	(GA)_8_TC	53.6°	2	100, 400
21.	UBC842	(GA)_8_TG	53.6°	2	200, 600
22.	UBC847	(CA)_8_AC	50°	3	100–500
23.	UBC848	(CA)_8_AG	50°	2	100, 500
24.	UBC853	(TC)_8_AT	50°	3	100–600
25.	UBC856	(AC)_8_CA	50°	2	300, 700
26.	UBC858	(TG)_8_AT	50.8°	1	300
27.	UBC859	(TG)_8_AC	52.2°	2	300, 500
28.	UBC870	(TGC)_6_	51.2°	2	100, 500
29.	UBC871	T(ATT)_6_	51.2°	3	100–600
30.	UBC872	(GATA)_4_	51.2°	3	200–700

### Application of the developed droplet-vitrification cryopreservation method to eleven other Jojoba genotypes

**Fig. 5:**
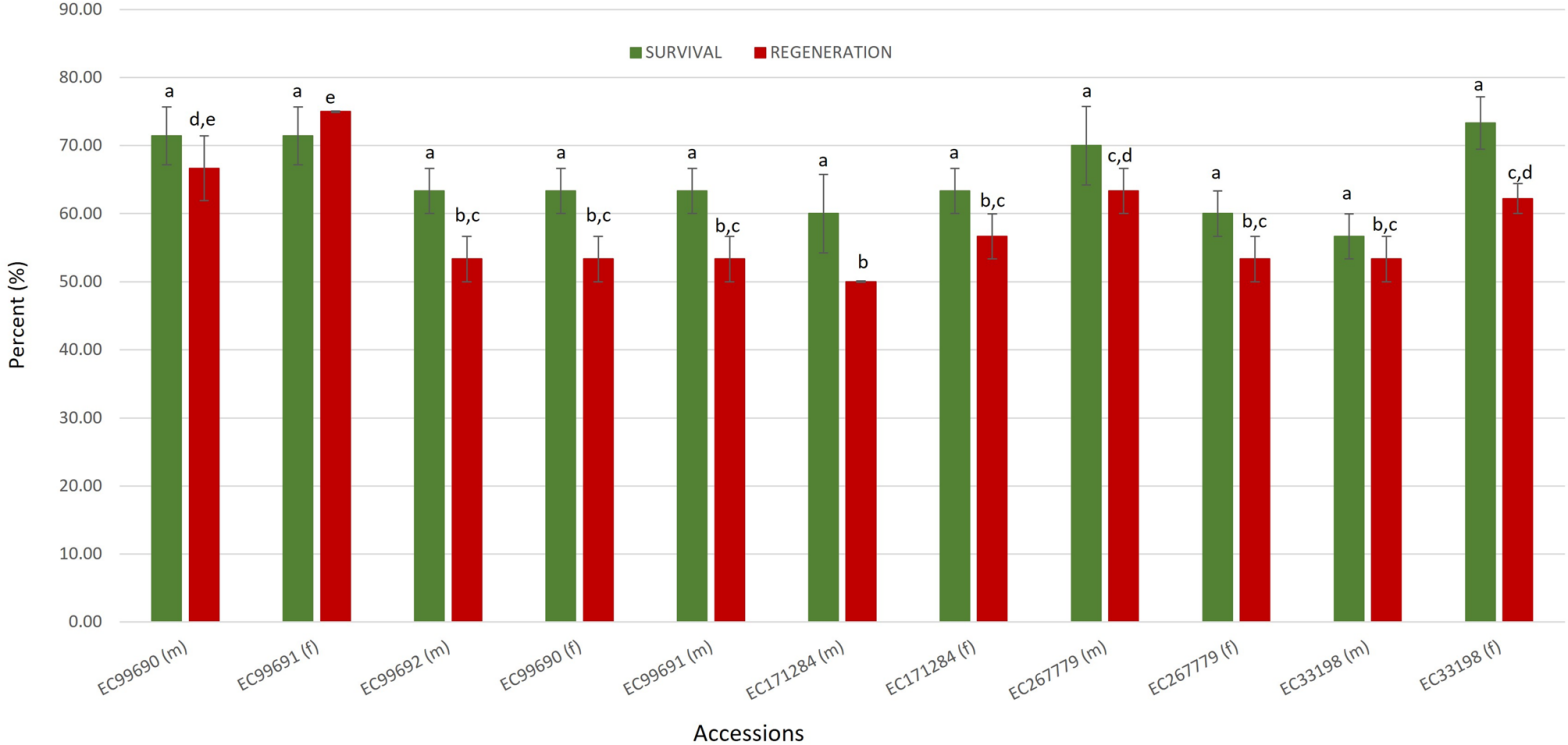
Post-thaw survival and regeneration rates of eleven jojoba accessions cryopreserved with droplet-vitrification. Data is presented as mean ± SE. Significant differences (*p* ≤ 0.05) are presented by different alphabets as analysed by DMRT. (m) and (f) following the accession number indicates male and female plants, respectively.

The droplet vitrification protocol developed above was tested in eleven other jojoba accessions. High post-thaw survival (85.71%) in accession EC 99,690 (male) and regrowth (75%) in accession EC 99,691 (female) were observed in the tested accessions, with a mean survival and regrowth rates of 69.76 and 53.31%, respectively, for the eleven accessions (Fig. 5). All the cryopreserved plants showed morphologies similar to their respective control plants. Hence, using the developed protocol all the accessions of jojoba were Cryobanked in the In Vitro Base Genebank of ICAR-NBPGR.

## Discussion

*Simmondsia chinensis* (jojoba) one of the most important commercial crops, is cultivated in marginal lands across the globe on soils were not much plantation is possible. It is a polyploid, dioecious, extremely heterogeneous perennial shrub producing highly heterozygous seeds. The many characteristics of jojoba such as its long-life span of over 100 years; its seed oil that is non-perishable, highly stable and versatile; disease, pest and drought resistance and growth in arid climates make it an inherently sustainable, renewable source of raw material for industry and land conservation that needs to be conserved for future sustainable growth. However, very little work has been done on its conservation. Conservation through seeds is challenging as jojoba is a dioecious species, hence, requiring conservation of both male and female plants separately. This is further compounded by the possibility of genetic heterogeneity within the seed lots requiring proper sampling before conservation to ensure no loss of genetic variability. As conservation of elite lines is important, medium-term conservation has been applied in case of jojoba^13–15^, but no long-term conservation strategy has yet been developed. The current study was taken up to develop a widely applicable, long-term conservation strategy for jojoba using the droplet vitrification method of shoot tip cryopreservation. Using this protocol, 50–76.13% of post-thaw shoot regrowth was observed, across twelve jojoba accessions. Parameters including sucrose preculture duration and cryoprotectant type, its duration and temperature were optimized to arrive at the most suited, efficient and reproducible protocol.

Optimal sucrose preculture is one of the key factors governing a successful cryopreservation protocol. In most cryopreservation protocols, shoot tips are precultured on high sucrose enriched medium to induce tolerance to dehydration and subsequent freezing^22^. It is known that accumulation of sugars slowly decreases water content of cells, thereby increasing membrane stability when cells are subjected to dehydration^23^. Metabolites such as endogenous sugars, polypeptides, abscisic acid and proline accumulate in cells on high sucrose preculture help improving the desiccation stress tolerance of cells^24^. Sugars also help in maintain the liquid crystalline state of membrane bilayers along with stabilizing proteins^25^. Below 20% fresh water level in cells, sugars act by replacing the hydration shell forming hydrogen bonds with different cellular macromolecules, and hence prevent protein denaturation and phase transition in membranes^26^. In the current study the duration (ranging from 1 to 5 days) of preculture on 0.3 M sucrose enriched medium was studied. It was observed that preculture for 2 days was optimal for highest post-thaw regrowth of cryopreserved jojoba shoot tips, which is in agreement with studies on cryopreservation of various other crops^27–32^. In addition, it was found that shoot regrowth decreased with increase in preculture duration, indicating the sensitivity of shoot tips to hyperosmosis^33^.

Another factor for governing the survival of cells and tissue during freezing in LN and recovery thereafter is the ability to avoid cell damage and maintain viability. The major contributing factor to cellular damage is ice crystallization during the cooling process, which can be avoided by treating the cells with suitable cryoprotectants before immersing them in LN^34^. PVS2 is one of the most commonly used cryoprotectant solutions. PVS2 replaces the cellular water along with altering the freezing behaviour of remaining intracellular water preventing dehydration induced injurious cell shrinkage^35^. However, its higher concentrations have been observed to have cytotoxic effects^35^, therefore, the exposure duration of cells to PVS2 along with its exposure temperature need to optimised when devising any cryopreservation strategy^36,37^. In the present work, the highest post-thaw survival and regrowth were observed when shoot tips were treated with PVS2 for 30 min. While exposure for shorter durations was found to be ineffective for optimal shoot regrowth, increasing the exposure duration beyond 30 min showed a detrimental effect. The optimal PVS2 exposure time varies with plant species, explant used and cryopreservation technique; however, 30 min exposure has been found to be suitable in crops such as thyme, Citrus, chrysanthemum, Stevia, Dahlia, hops^37–42^. Further, chilled PVS2 has been used in several crops to enhance post-thaw survival as lowering the temperature is believed to reduce its cytotoxicity^43–45^. Hence, the effect of chilled PVS2 on post-thaw survival and regrowth was studied. However, it was observed that in case of jojoba, chilled PVS2 did not improve the post-thaw regrowth of shoot tips.

PVS3, which does not contain dimethyl sulfoxide (DMSO), has also been increasing used in droplet vitrification protocols instead of PVS2 owing to the cytotoxic effects of the latter. PVS3 was first used in *Asparagus officinalis* L. cryopreservation^46^ and since then has been used by several research groups in cryopreservation of such species which show sensitivity to PVS2^27,47,48^. Taking these observations into consideration experiments were conducted to study the effect of PVS3 on shoot tip cryopreservation of jojoba. It was observed that PVS3 did not offer any significant advantage in post-thaw survival over PVS2 in this case. Further, the post-thaw survival and regrowth in case of using chilled PVS3 as well as PVS3 at room temperature was significantly lower that the survival and regrowth at PVS2. It has been concluded that the optimal cryoprotectant solution concentration and type widely species specific and the treatment conditions are dependent on shoot tip size and preculture and pretreatment conditions^49,50^.

As during the process of cryopreservation shoot tips have to under several stress including osmotic dehydration, expose to high viscosity chemicals, cryo injury due to expose to ultra-low temperatures as well in vitro manipulations, they are prone to under various genetic variations such as gene mutations, chromosomal rearrangements, activation of transposable elements etc^51^. Hence it is imperative to confirm the genetic homogeneity of plants regenerated after cryopreservation to ensure that the cryopreservation protocol is robust and does not induce any variations. ISSR markers have been used in various studies for the assessment of genetic stability of cryopreserved plants as they are hyper-variable and highly polymorphic multi-locus markers having a probability of detecting intra-genome variability^52–54^. No variation was detected between the tested in the present study too, indicating that the droplet-vitrification protocol generally does not induce genetic variation in regenerated plants.

It is a challenge to arrive at a generic protocol suitable for cryopreservation of a large number of accessions as genotype dependent variation in post-cryo survival is well documented^42^,^55–58^. Our results show that the developed protocol was suitable for cryopreservation of 12 different accessions, with post-thaw regrowth ranging from 50 to 76.13%. The variable response may be attributed to a combination of factors including the age and condition of source plant material as well as the different stress conditions the explant undergoes during cryopreservation. Although, in the present study the differences between the accessions in terms of post-thaw survival were observed, however, as greater than 50% regrowth was recorded in all the accession, we can conclude that the developed protocol can be used for conservation of in vitro raised jojoba germplasm.

Volk et al. (2017)^59^ developed a decision matrix to help decide the number of samples required for cryobanking any accession in a genebank. According to them, having one-two control vials for thawing is necessary to estimate the number of shoot tips that would remain viable after cryopreservation. Further, they estimated that with a 40% viability at 95% confidence, keeping 100 explants would result in 32 viable shoot tips. In the present study, viability of over 50% was achieved in all the accessions, and hence storing 3 sets of ten vials each, with each vial having 10 explants would sufficiently meet the purpose of cryobanking. This further emphasizes the efficacy and reproducibility of the developed droplet vitrification protocol.

## Conclusion

A simple yet efficient droplet vitrification based cryopreservation protocol has been developed in jojoba collections. Jojoba responds well to cryopreservation, and high post-thaw survival and regeneration can be achieved by preculturing the shoot tips on 0.3 M sucrose for 2 days, before treating them with loading solution for 20 min, followed by PVS2 exposure of 30 min and then plunging in LN for cryopreservation. The protocol has been tested for its reproducibility on twelve different accessions. To the best of our knowledge, this is the first report on cryopreservation of jojoba and can be applied for long term conservation of valuable germplasm which will act as a resource for the future, in light of the changing environmental and climatic conditions.

## Methods

### Plant material

Twelve accessions of jojoba maintained as in vitro cultures in the In Vitro Genebank of ICAR-NBPGR were used in the present study. One accessions, namely EC99692 female, was used for optimizing the key parameters for developing the droplet vitrification protocol. The other accessions were subsequently used for validation of the optimized protocol.

### Maintenance of in vitro stock cultures

In vitro stock cultures were maintained on the maintenance medium comprising of 4.84µM benzyl aminopurine (BAP) (Sigma-Aldrich, St. Louis, USA) in MS medium^60^ containing 30 g/l sucrose and solidified with 8 g/l agar (both from (Hi-Media^®^ Laboratories, Mumbai, India). After adjustment of the medium pH to 5.8, the medium was autoclaved at 121 °C for 20 min. The cultures were maintained at Standard Culture Room conditions at 25 ± 2 °C under a 16/8 hr photoperiod and light intensity of 40 µmol m^− 2^s^− 1^ maintained by cool white, fluorescent tubes (Philips, Mumbai, India). Cultures were subcultured onto fresh medium every four weeks.

### Droplet-vitrification protocol

Shoot tips (approximately 2 mm) were excised from eight-week-old in vitro cultures multiplied on the maintenance medium (Fig. 1a). Explants were then precultured on semisolid preculture medium (PCM) comprising of MS medium containing 0.3 M sucrose for different time durations depending on experiment. Following preculture, explants were transferred to petri plates containing loading solution (LS) comprising of 2.0 M glycerol (Sigma-Aldrich) and 0.4 M sucrose in liquid MS medium with pH 5.8^57^ for 20 min. For cryoprotection, the explants were then transferred to PVS2/ PVS3 for 10 to 50 min at room temperature/0°C. PVS2 comprised of liquid MS medium supplemented with 0.4 M sucrose, 30% (w/v) glycerol, 15% (w/v) ethylene glycol, and 15% (w/v) dimethyl sulfoxide (Sigma-Aldrich) at pH 5.8^61^; while PVS3 was made up of liquid MS medium supplemented with 50% (w/v) sucrose and 50% (w/v) glycerol in liquid MS medium, pH 5.8^46^. After completion of cryoprotection, explants were exposed to liquid nitrogen (LN) by placing ten shoot tips in a drop of PVS2/PVS3 on a pre-sterilized aluminium foil strip and directly plunging the strip into LN. Frozen foil strips were transferred to cryovials (1.8 ml) suspended in LN in polycarbonate freezer boxes placed in thermocol ice box. During protocol optimization, explants were stored in LN for at least 1 h before proceeding to rewarming. For rewarming, foil strips containing the explants were removed from LN and immediately immersed in rewarming solution containing liquid MS medium with 1.2 M sucrose (pH 5.8) and incubated for 15 min at room temperature. Cryopreserved shoot tips were then placed on sterile filter paper placed on preculture medium and incubated overnight in dark. Thereafter, shoot tips were placed on the regeneration medium (MS + 4.97µM BAP + 0.28 µM GA_3_) and incubated in dark for three to five days. Explants were then subsequently shifted to light conditions under standard culture room conditions and observed for survival and recovery.

Shoot tips were considered to be surviving when they showed green tissue after seven days of cryopreservation, while shoot tips were counted as regenerating when they regenerated into proper shoots after four-six weeks of post-thaw culture.

### Experiment 1: effect of preculture duration on post-thaw recovery

The excised shoot tips were precultured on PCM for 1, 2, 3, 4 and 5 days under standard culture room conditions. The shoot tips were then treated with LS for 20 min, followed by PVS2 vitrification. The rest of the procedure was as described above.

### Experiment 2: effect of PVS2 treatment duration and temperature on post-thaw recovery

The excised shoot tips were precultured on PCM for 1, 2, 3, 4 and 5 days under standard culture room conditions, followed by LS treatment for 20 min. Shoot tips were then dehydrated with PVS2 for 10, 20, 30, 40 and 50 min at room temperature, before plunging them in LN. The procedure for rewarming and recovery was followed as described above.

In a separate set of experiment, excised shoot tips were treated with chilled PVS2 (at 0 °C) for 10, 20, 30, 40 and 50 min after preculture on PCM and LS treatment of 20 min and then plunged in LN. Shoot tips were revived as described earlier.

### Experiment 3: effect of PVS3 duration and temperature on post-thaw recovery

To study the effect on PVS3 on post thaw recovery (different dehydration regimes both at room temperature and 0 °C), the experiments described above were repeated, using PVS3 instead of PVS2.

### Assessment of genetic stability

Post-thaw regenerated plants were assessed for their genetic stability using ISSR markers. Genomic DNA was extracted from fresh young leaves of six-month old in vitro stock cultures, controls referred to as -LN (plants vitrified with PVS2 for 30 min but not frozen in LN) and randomly selected post-thaw regenerated plants (+ LN), i.e. plants vitrified using PVS2 for 30 min followed by freezing in LN using the CTAB method^62^. 30 ISSR (UBC Primer Set No. 9, Vancouver, BC, Canada) markers were selected for analysis (Table 1). PCR amplification was carried out in 10 µL reaction mixtures containing 1X reaction mixture (One PCR™, GeneDireX Inc. USA), 10 µM primer and 40 ng template DNA in a thermal cycler (Gene Pro, Hangzhou Bioer Technology Co., China) with the thermal profile as: initial denaturation at 94 °C for 5 min followed by 38 cycles of denaturation at 94 °C (10 s), primer annealing at Tm varying as per primer (30 s) and primer extension at 72 °C (65 s), and a final extension (72 °C) for 10 min. Results were analysed on 2.5% agarose gels and photographed on a Gel Documentation System (GenoSens 2100, Clinx Science Instruments Co., China). Size of amplified products was analysed by co-electrophoresis with a 100 bp DNA marker. Only primers showing good quality compact bands were used for further analysis.

### Application of developed protocol to other Jojoba accessions

The optimized protocol was further validated on eleven other jojoba accessions (both male and female plants of EC99690, EC99691, EC171284, EC267779, EC33198 and male plants of EC99692). Shoot tips excised from eight-week-old cultures maintained on maintenance medium were precultured on PCM for 2 days, treated with LS for 20 min, followed by vitrification with PVS2 at room temperature for 30 min and then frozen in LN. Shoot tips were then rewarmed as described earlier and data on post-thaw survival and regrowth was recorded.

### Data analysis

All experiments were carried out in using Completely Randomized Design (CRD) repeated thrice with ten explants in each replication. Shoot tips dehydrated with the cryoprotectants (PVS2/PVS3) but not exposed to LN were considered as control (-LN), while cryopreserved shoot tips were designated as + LN. Data was generated as percentage and transformed to arc sine values before analysis. Data was analysed with one-way ANOVA test (*p* < 0.05) and significant difference between means was assessed using the Duncan’s Multiple Range Test (DMRT). Results were presented as mean with standard error (SE). All analysis was performed using SPSS statistics version 22.0 software package.

## Data Availability

All data generated or analysed during this study are included in this published article.
